# Cardioprotective effect of isorhamnetin against myocardial ischemia reperfusion (I/R) injury in isolated rat heart through attenuation of apoptosis

**DOI:** 10.1111/jcmm.15267

**Published:** 2020-04-19

**Authors:** Yan Xu, Chun Tang, Shengyu Tan, Juan Duan, Hongmei Tian, Yu Yang

**Affiliations:** ^1^ Department of Geriatrics the Second Xiangya Hospital Central South University Changsha PR China; ^2^ Department of Nephrology Center of Nephrology and Urology the Seventh Affiliated Hospital Sun Yat‐sen University Shenzhen PR China

**Keywords:** apoptosis, cardioprotective effect, isorhamnetin, myocardial ischaemia reperfusion (I/R) injury, oxidative stress

## Abstract

In this study, we investigated the effects of isorhamnetin on myocardial ischaemia reperfusion (I/R) injury in Langendorff‐perfused rat hearts. Isorhamnetin treatment (5, 10 and 20 μg/mL) significantly alleviated cardiac morphological injury, reduced myocardial infarct size, decreased the levels of marker enzymes (LDH and CK) and improved the haemodynamic parameters, reflected by the elevated levels of the left ventricular developed pressure (LVDP), coronary flow (CF) and the maximum up/down velocity of left ventricular pressure (+dp/dt_max_). Moreover, isorhamnetin reperfusion inhibited apoptosis of cardiomyocytes in the rats subjected to cardiac I/R in a dose‐dependent manner concomitant with decreased protein expression of Bax and cleaved‐caspase‐3, as well as increased protein expression of Bcl‐2. In addition, I/R‐induced oxidative stress was manifestly mitigated by isorhamnetin treatment, as showed by the decreased malondialdehyde (MDA) level and increased antioxidant enzymes activities of superoxide dismutase (SOD), catalase (CAT) and glutathione peroxidase (GSH‐Px). These results indicated that isorhamnetin exerts a protective effect against I/R‐induced myocardial injury through the attenuation of apoptosis and oxidative stress.

## 1 INTRODUCTION

Acute myocardial infarction (AMI) is a leading cause for the death of coronary heart disease and became a serious public health concern with high mortality, morbidity and disability rates worldwide.^1^ Theoretically, in‐time myocardial reperfusion to the ischaemic myocardium is one of the most effective strategies for the treatment of AMI. In most circumstances, restoring blood supply to the damaged myocardial tissue can prevent ischaemia‐induced heart damage; however, in the same time of saving lives, reperfusion itself may further aggravate the myocardial cell death and induce abnormal cardiac function, which is known as ischaemia/reperfusion (I/R) injury.^2^, ^3^ Such inevitable secondary injury can seriously exacerbate patient's pathological disorders, such as myocardial stunning, transient mechanical dysfunction, reperfusion arrhythmias, cell death and other disorders.^4^ Therefore, in the clinical setting, timely myocardial I/R injury treatment will definitely improve therapy outcome for patient with AMI.

Although the phenomenon of myocardial I/R injury existed for many decades, scientists never stop exploring the pathophysiology mechanisms behind myocardial I/R injury and developing novel drugs to treat or alleviate it.^5^ Myocardial I/R injury is an intricate pathophysiological process that involves multifactorial mechanisms, such as increased inflammatory response, intracellular calcium overload, oxidative stress, myocardial necrosis and apoptosis.^6^, ^7^, ^8^ Among these contributors, a substantial body of evidence has suggested that apoptosis plays a critical role in myocardial I/R injury, which impairs cardiac function.^9^ Apoptosis is a continually occurring programmed cell death that maintains tissue homoeostasis, and the importance of apoptosis contributed to myocardial I/R injury has been well documented by many research groups.^10^, ^11^ Long periods of myocardial ischaemia result in an increase of necrosis, but paradoxically, reperfusion leads to an enhancement in apoptosis.^12^ Myocardial apoptosis accelerates the development of necrosis which might determine the degree of myocardial injury. More importantly, oxidative stress during I/R‐induced myocardial damage is closely associated with cell apoptosis^13^ and usually occurs when the generation of ROS prevails over antioxidant system function in the organism.^14^, ^15^ Excessive ROS can cause damage to the structure of myocardial mitochondria and then trigger mitochondrial‐mediated cell apoptosis.^16^, ^17^ It is evident that suppressing myocardial oxidative stress and cell apoptosis may attenuate I/R‐induced cardiac injury^18^ and can be considered as a pivotal target for therapeutic intervention for this diseases. Therefore, seeking antioxidant and anti‐apoptotic agent from plants is necessary for the treatment of myocardial I/R injury.

Natural flavonoids, which are natural polyphenols universally found in herbal drugs and foods, have attracted considerable attention for their biological and physiological importance. Interestingly, increasing epidemiological studies demonstrated an inverse correlation between flavonoids intake and heart disorders incidence.^19^, ^20^, ^21^ Therefore, flavonoids have the most potential to be developed as safe therapeutics for cardiovascular disease. Isorhamnetin, a naturally distributed flavonoid in the sea buckthorn, *Oenanthe javanica* and *Ginkgo biloba* L.,^22^ has been confirmed that it can attenuate atherosclerosis, protect cardiomyocytes against oxidation^23^, ^24^ or anoxia/reoxygenation,^25^ ameliorate cardiac hypertrophy^26^ and rescue rat ventricular myocytes from I/R injury in vitro via repressing apoptosis.^27^ These findings highlight the importance of antioxidant and anti‐apoptotic properties of isorhamnetin on the therapy of cardiovascular diseases. However, whether isorhamnetin has the protective effect against myocardial I/R injury via the inhibition of oxidative stress and apoptosis have yet to be fully uncovered. With this in mind, in this study, we intend to examine the effect of isorhamnetin treatment on myocardial I/R injury in isolated rat heart and investigated the underlying mechanism underpinning this effect.

## 2 MATERIALS AND METHODS

### 2.1 Materials and Reagents

Isorhamnetin (purity >98%), 2,3,5‐triphenyltetrazolium chloride (TTC) and all of the other reagents were obtained from Sigma‐Aldrich. The malondialdehyde (MDA) content and the activity of Lactate dehydrogenase (LDH, sensitivity: 7.81 mU/mL and CV% <8%) and creatine kinase (CK, sensitivity: 0.039 mU/mL and CV% < 8%), catalase (CAT), glutathione peroxidase (GSH‐Px) and superoxide dismutase (SOD) were purchased from Nanjing Jiancheng Bioengineering Institute. The BCA protein assay kit and enhanced chemiluminescence (ECL) reagent were purchased from Pierce. An In Situ Cell Death Detection kit was from Roche.

### 2.2 Animals and ethics statement

Adult male Sprague‐Dawley (SD) rats weighing 250 ± 20 g were purchased from Animal Experimental Center of Central South University and were housed under laboratory pathogen‐free conditions with a 12:12‐hour light‐dark cycle at 25 ± 2°C and 50% ± 15% humidity. The animals had free access to a regular pellet diet and tap water. All animal experimental procedures were conducted in adherence with the guidelines approved by the Animal Care and Use Committee of the Second Xiangya Hospital, Central South University in Changsha, China.

### 2.3 Induction of I/R and experimental design

The establishment of I/R model was performed as previously described by Jiang et al, with some modifications.^28^ All rats were allowed to acclimatize the experimental environment for one week and then anaesthetized with pentobarbital sodium (100 mg/kg) via intraperitoneal injection (i.p.), followed by i.p. injection of 250 U/kg heparin to avoid coagulation. After that, the hearts were quickly removed and immediately arrested in 40 mL of ice‐cold Krebs‐Henseleit (K–H; pH 7.4) perfusion buffer (composition in mmol/L: NaCl 118, KCl 4.7, CaCl_2_ 2.5, glucose 11.0, MgSO_4_ 1.2, KH_2_PO_4_ 1.2, NaHCO_3_ 25.0). Finally, the excised hearts were cannulated through the ascending aorta on a Langendorff apparatus for retrograde perfusion with a K–H buffer, which was bubbled with 95% O_2_ and 5% CO_2_ to maintain a pH 7.35‐7.45 under constant perfusion pressure of 75mmHg at 37°C. A water‐filled latex balloon connected with a pressure transducer was inserted into the left ventricular cavity via the left auricle to continuously record left ventricle pressure until the left ventricular enddiastolic pressure (LVEDP) stably kept between 5 and 12 mmHg.

**FIGURE 1:**
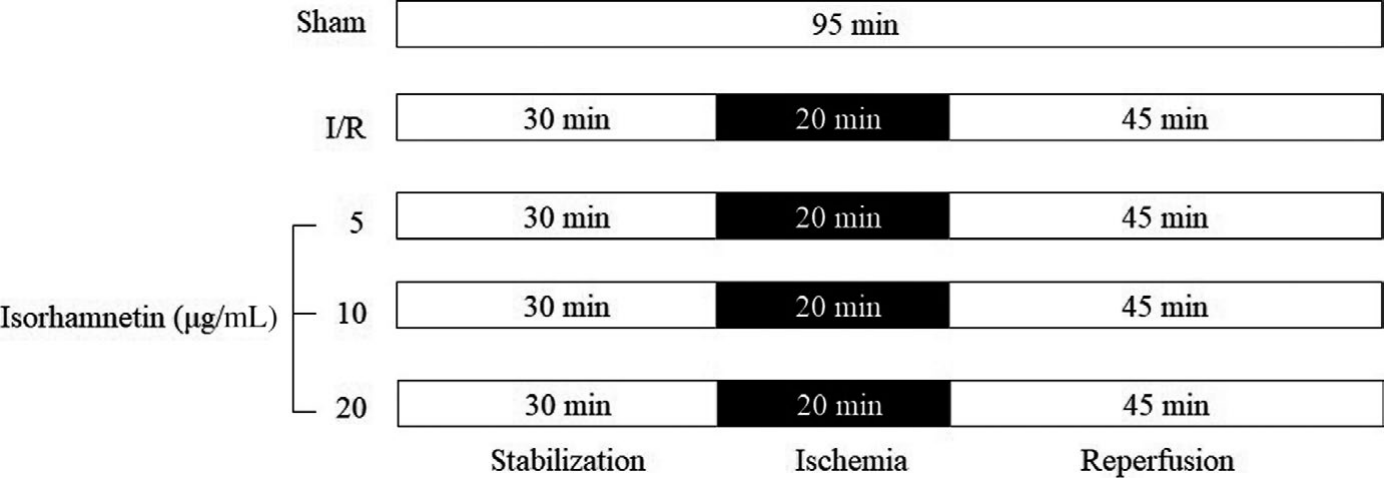
Experimental protocol for myocardial ischaemia/reperfusion (I/R) stimulation and isorhamnetin addition in perfused rat heart

The isolated mouse hearts were randomly assigned to 5 groups (10 in each group) based on simple randomization method with flipping a coin: control group (sham), model group (I/R) and isorhamnetin groups (treated with 5, 10 and 20 μg/mL). The sham control hearts were continuously stabilized for 95 minutes. I/R group hearts were stabilized for 30 minutes and then subjected to zero‐flow global ischaemia for 20 minutes before 45 minutes of reperfusion. Isorhamnetin‐treated groups were performed the same procedures as I/R control, except that reperfusion was performed with isorhamnetin‐containing K–H buffer for 45 minutes (Figure 1).

### 2.4 Assessment of cardiac function

During 45 minute's reperfusion, the left ventricular developed pressure (LVDP), heart rate (HR), coronary flow (CF), the maximum up velocity of left ventricular pressure (+dp/dt_max_) and the maximum down velocity of left ventricular pressure (−dp/dt_max_) were continuously measured using a computer‐based data acquisition system (PC PowerLab with Chart 5 software, 4S AD Instruments) according to the manufacturer's instructions. The coronary effluent from the apex of the heart was selected to monitor CF by timed collection o at 15, 30 and 45 minutes intervals with a constant pressure of 80 mm H_2_O throughout the experiment.

### 2.5 Measurement of myocardial infarct size and cardiac marker enzymes

The myocardial infarct size was measured by TTC staining method as previously described.^29^ After 45 minutes of reperfusion, the hearts from three mice in each group was rapidly excised and frozen at −70°C until being sliced transversally into 2 mm thick axial sections. These sections were soaked in 1% TTC (pH 7.4) away from light for 20 minutes at 37°C and then subsequently fixed in 4% paraformaldehyde for 24 hours. TTC‐positive staining area in deep red colour represents the viable myocardial tissue, while the pale white section means nonviable myocardium. The percentage of infarct area size at risk was assessed digitally using Image J software as previously described.^30^

After the experiment, the levels of LDH and CK in the perfusate from the other left mice (n = 7) in each group were spectrophotometrically measured to evaluate the degree of cardiac cell death using respective cytotoxicity detection commercial kit in accordance with the manufacturer's protocols.

### 2.6 Histopathology analysis

For histological analysis, a portion cardiac tissues from each group (n = 7) were fixed in 4% paraformaldehyde solution at 4°C overnight for embedding in paraffin. The paraffin‐embedded sections (5 μm) were stained with haematoxylin and eosin (H&E), followed by histopathological examination under a light microscope at 400× magnification.^31^

### 2.7 Measurement of myocardial apoptosis

At the end of experiment, a portion of myocardial tissue from various groups (n = 7) was fixed with 4% formalin, embedded in paraffin, and then sectioned at 5 μm thickness, as described previously.^31^ For apoptosis determination, a terminal deoxynu‐cleotidyl transferase dUTP nick end labelling assay (TUNEL) assay was performed using an In Situ Cell Death Detection kit according to the manufacturer's recommendations. After TUNEL staining, the stained specimen was then added with haematoxylin to stain myocardial cell nuclei, followed by images observation under a light microscope at 200× magnification. For each paraffin section, three random, high‐power fields were selected and the average number of TUNEL‐labelled nuclei were counted and quantified. Apoptotic index was estimated as the percentage of stained cells with respect to the total number of myocytes.

### 2.8 Assessment of oxidative stress

A portion of heart tissues in different group (n = 7) were lysed and homogenized with the appropriate phosphate buffer using a microcentrifuge tube homogenizer, followed by centrifugation (5000 rpm, 15 minutes) to yield their individual supernatants. The MDA content and the activity of CAT, GSH‐Px and SOD were measured in cardiac homogenates supernatants using respective specific assay kits according to the manufacturer's protocol.

### 2.9 Western blot analysis

The expression levels of Bax, Bcl‐2 and cleaved‐caspase‐3 protein extracted from myocardial tissues were measured using Western blot, as documented by Yao et al^32^ Briefly, after reperfusion, the same regions of myocardial tissue from each group (n = 7) were crushed and lysed in RIPA lysis buffer supplemented with protease and phosphatase inhibitors, and then homogenized under 4°C. The resulting samples were kept on ice for 20 minutes, after which the lysate was subject to centrifugation (12,000 *g* for 10 minutes) to yield the total proteins. The content of this total protein was quantified using the BCA protein assay kit. Each sample with a total of 30 μg proteins was separated by 12% sodium dodecyl sulphate polyacrylamide gel electrophoresis (SDS‐PAGE) and then transferred to nitrocellulose membranes, followed by blocking with 5% non‐fat milk at 30°C for 30 minutes. Thereafter, the membranes were incubated overnight (4°C) with primary antibodies against Bcl‐2 (1:1000), Bax (1:500), cleaved‐caspase‐3 (1:500) and β‐actin (1:25,000) and then probed with appropriate horseradish peroxidase‐conjugated secondary antibodies for 0.5 hour at room temperature. The protein bands were detected using a chemiluminescence detection kit and quantified with Image J software (National Institutes of Health, Bethesda, MD) by normalizing to β‐actin.

### 2.10 Statistical analysis

All data are expressed as the mean ± standard deviation (SD) and evaluated by a one‐way or two‐way mixed analysis of variance (ANOVA) followed by the post hoc Tukey test. For all analyses, GraphPad Prism software was used (GraphPad Software, Inc, La Jolla, CA), and a value of *P* < .05 was considered to be statistically significant.

## 3 RESULTS

### 3.1 Isorhamnetin reduced myocardial structure injury, myocardial infarct size and myocardial enzyme activities in isolated rat hearts subjected to I/R

**FIGURE 2:**
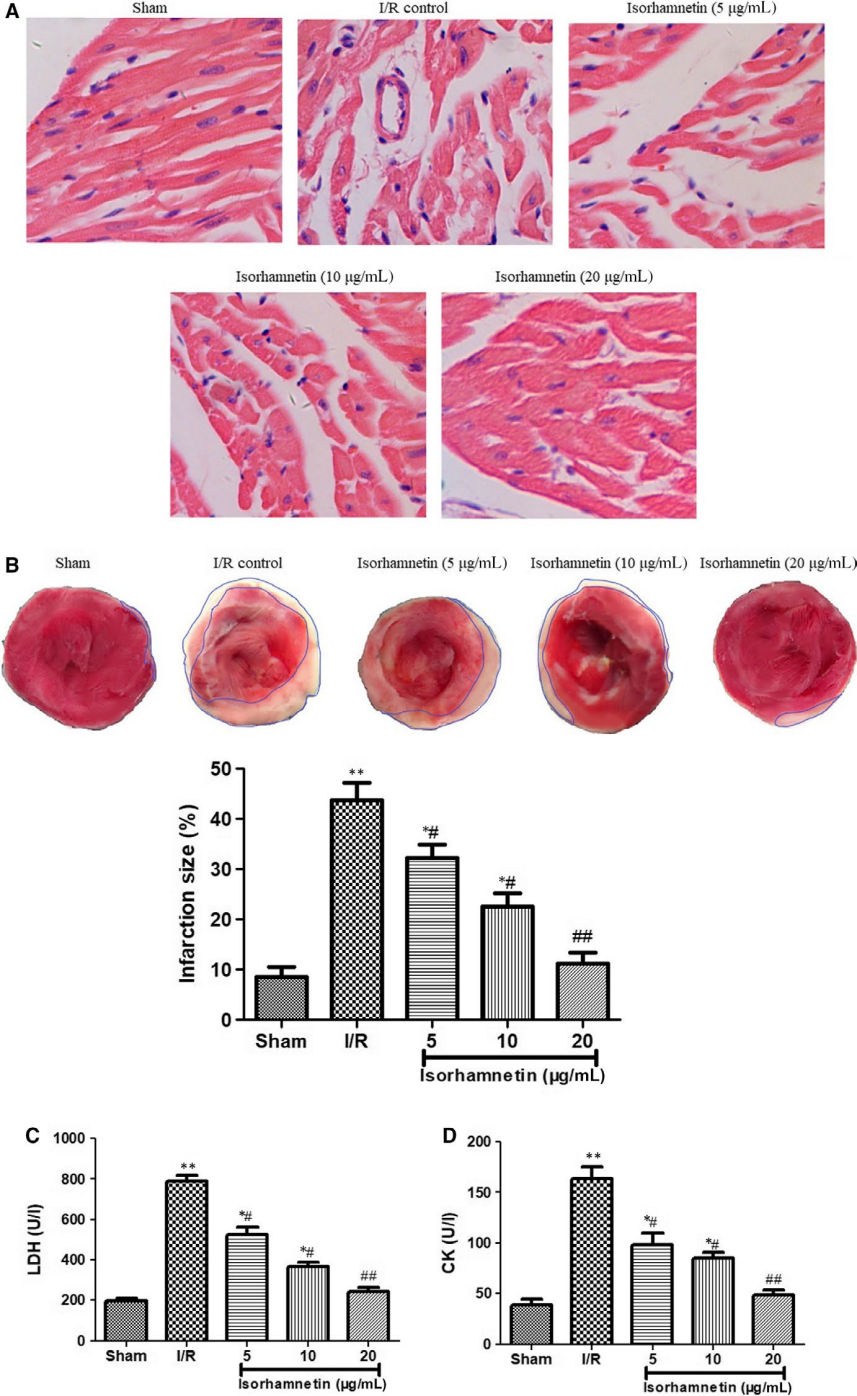
Isorhamnetin attenuated myocardial I/R injury in isolated rat hearts. (A) Representative photomicrographs of HE staining in the rat hearts (×200 magnification, scale bar 200 μm) (B) Myocardial infarct size was measured by TTC staining. The myocardial infarct size was expressed as the percentage of infarct relative to the total area. (C) The LDH levels in coronary effluent were measured by commercial assay kits. (D) The CK levels in coronary effluent were measured by commercial assay kits. The data were expressed as mean ± S.D (n = 7). ^#^ *P* < .05 and ^##^ *P* < .01 compared with the I/R group; *P < .05 and **P < .01 compared with the sham group

The myocardium histopathological change in different groups was examined by H&E staining (Figure 2A). The myocardial cells from sham operation rats showed well‐arranged cell architecture and intact muscle fibres, while I/R injury lead to aggravated myocardium damage, including irregularly arranged muscle fibres, widespread necrosis, cell oedema and the blurred boundaries between cells. However, treatment with isorhamnetin at 5, 10 and 20 μg/mL significantly reversed I/R‐induced pathological changes in isolated rat hearts tissues when compared with I/R group. The arrangement of myocardial fibres was relatively neat and tight, and the necrosis extent of cardiomyocytes was significantly reduced.

In consistent with the myocardial morphological alteration, I/R operation caused a significant increase of infarction size manifested by the more pale white areas as compared with the sham group. Digital computation of the TTC staining displayed that isorhamnetin treatment (5, 10 and 20 μg/mL) markedly reduced the infarction area of the isolated rat hearts in a dose‐dependent manner (Figure 2B).

After 20 minutes of ischaemia followed by 45 minutes of reperfusion, the coronary effluent samples were collected to measure the release of LDH and CK as degree of myocardial injury (Figure 2C,D). The serum CK and LDH level in the I/R group was notably higher than those in sham control (*P* < .01). However, sorhamnetin treatment (5, 10 and 20 μg/mL) resulted in a visible reduction in these two myocardial enzyme activities with relative to those of I/R groups (*P* < .05 or *P* < .01).

### 3.2 Isorhamnetin improved cardiac function in isolated rat hearts subjected to I/R

To verify whether isorhamnetin has a protective effect on IR rats, haemodynamic parameters (LVDP, ±dp/dtmax, HR and CF) were continuously recorded during the experiments to evaluate the cardiac function using a computer‐based data acquisition system. As illustrated in Table 1, I/R treatment resulted in a remarkable reduction in the levels of LVDP, ±dp/dtmax and CF in isolated rat heart following reperfusion at 15, 30 and 45 minutes when compared to those in the shaml group (*P* < .01). There is not any statistical difference for HR between sham and I/R groups (*P* > .05). However, these haemodynamic function indexes were totally reversed by isorhamnetin treatment at three doses (5, 10 and 20 μg/mL) as comparison with I/R group (*P* < .05 or *P* < .01).

**TABLE 1:** Effect of isorhamnetin on cardiac function in rats subjected to I/R

Physical index	Reperfusion (%)		
	15 minutes	30 minutes	45 minutes
LVDP			
Sham	96.21 ± 8.65	95.75 ± 8.95	93.96 ± 8.64
I/R	48.68 ± 4.32^##^	45.82 ± 4.17^##^	44.01 ± 3.98^##^
Isorhamnetin			
5 μg/mL	63.54 ± 5.38*	66.87 ± 5.75*	68.04 ± 5.68*
10 μg/mL	76.54 ± 6.58**	77.85 ± 6.87**	78.52 ± 6.88**
20 μg/mL	85.25 ± 7.54**	87.36 ± 7.62**	88.45 ± 7.68**
+dp/dt_max_			
Sham	102.32 ± 9.54	103.21 ± 9.87	103.54 ± 9.62
I/R	53.21 ± 4.62^##^	48.54 ± 4.21^##^	47.42 ± 4.30^##^
Isorhamnetin			
5 μg/mL	65.54 ± 5.63*	65.21 ± 5.34*	67.21 ± 5.81*
10 μg/mL	75.21 ± 6.12**	75.78 ± 6.23**	76.58 ± 6.30**
20 μg/mL	82.01 ± 7.15**	83.65 ± 7.32**	85.21 ± 7.55**
−dp/dt_max_			
Sham	96.54 ± 8.59	96.21 ± 8.65	97.51 ± 8.88
I/R	53.91 ± 4.35^##^	53.20 ± 4.52^##^	52.00 ± 4.65^##^
Isorhamnetin			
5 μg/mL	63.54 ± 5.32*	65.35 ± 5.76*	66.68 ± 7.89*
10 μg/mL	75.21 ± 6.31*	76.33 ± 6.87*	78.54 ± 6.58**
20 μg/mL	81.20 ± 7.65*	82.65 ± 7.21**	83.54 ± 7.66**
HR			
Sham	97.55 ± 8.25	95.56 ± 8.64	96.38 ± 8.32
I/R	86.68 ± 7.65	87.54 ± 7.18	86.36 ± 7.95
Isorhamnetin			
5 μg/mL	88.95 ± 7.81	89.54 ± 7.86	88.88 ± 7.64
10 μg/mL	90.21 ± 8.12	91.85 ± 8.32	93.28 ± 8.51
20 μg/mL	93.89 ± 8.43	94.87 ± 8.62	94.81 ± 8.55
CF			
Sham	106.58 ± 9.27	107.21 ± 9.41	104.46 ± 9.68
I/R	56.33 ± 4.95^##^	53.12 ± 4.55^##^	52.54 ± 4.68^##^
Isorhamnetin			
5 μg/mL	68.54 ± 5.98*	70.22 ± 6.35*	71.31 ± 6.48*
10 μg/mL	75.65 ± 6.95*	76.21 ± 6.54*	77.26 ± 6.87**
20 μg/mL	79.64 ± 7.02**	82.24 ± 7.52**	83.05 ± 7.34**

Note

The data were expressed as mean ± S.D (n = 7).

^##^ *P* < .01 compared with the I/R group;

* *P* < .05 and

** *P* < .01 compared with the sham group.

### 3.3 Isorhamnetin attenuated myocardial apoptosis in isolated rat hearts subjected to I/R

**FIGURE 3:**
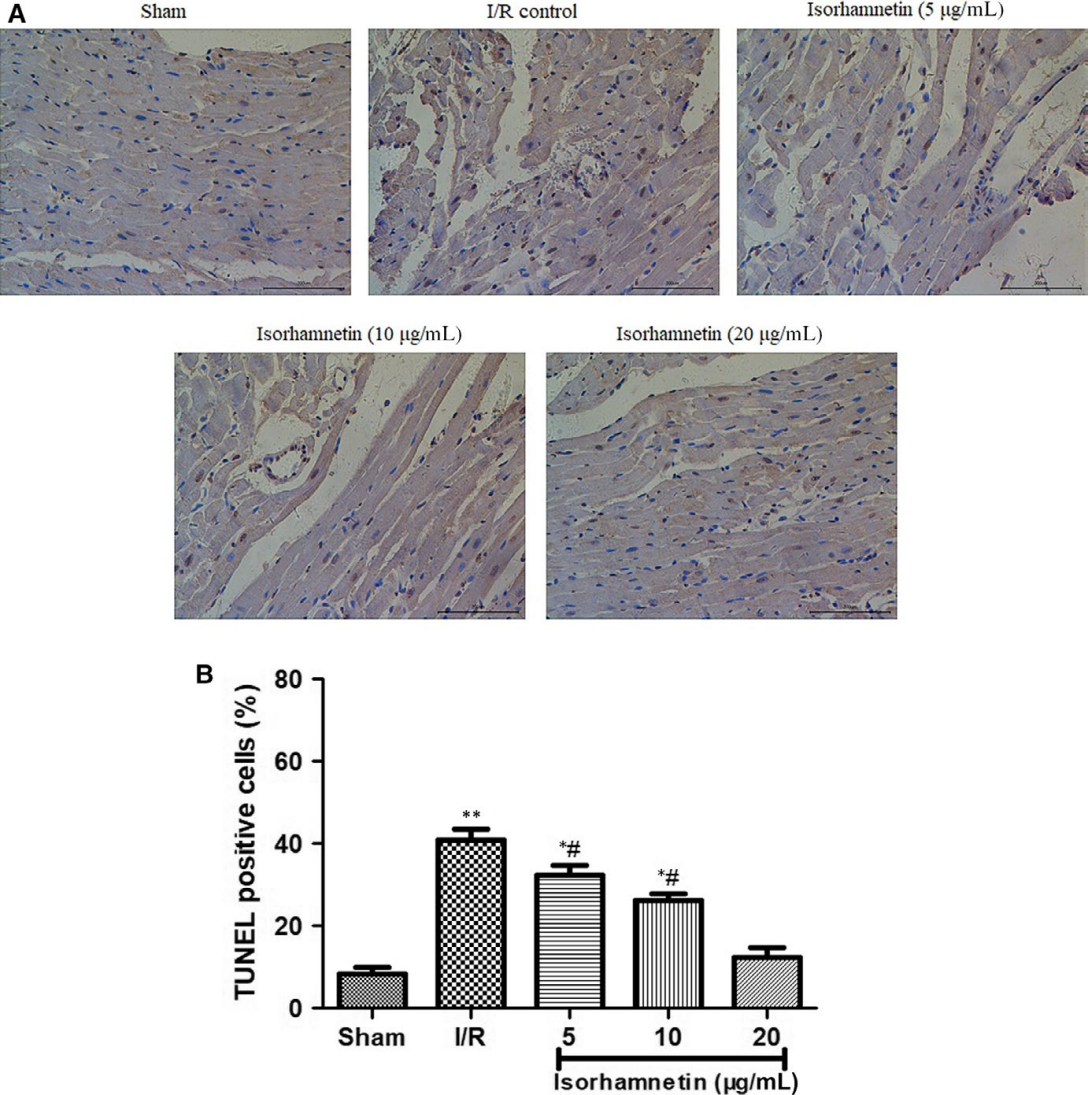
Isorhamnetin attenuated apoptosis in isolated rat hearts subjected to I/R. (A) Representative photomicrographs of TUNEL staining in the rat hearts (×200 magnification, scale bar 200 μm). (B) Quantitative results of TUNEL staining analysis. The data were expressed as mean ± S.D (n = 7). ^#^ *P* < .05 compared with the control group; *P < .05 and **P < .01 compared with the I/R group

Next, the influence of isorhamnetin on myocardial I/R‐induced cardiomyocyte apoptosis was confirmed by TUNEL staining. Cells with brown granules in the nucleus were considered as apoptotic cells. Under optical microscopy, this staining showed the more appearance of TUNEL‐positive cells with dark brown cell nuclei in the I/R group than that in the sham group; whereas this increase was changed to the opposite by isorhamnetin in a dose‐dependent way (Figure 3A). The apoptosis index in sham and I/R control is 41.06%±3.41% and 8.40% ± 0.92%, respectively. 5, 10 and 20 μg/mL of isorhamnetin co‐ reperfusion significantly reduced the percentage of TUNEL‐positive cells to 32.26% ± 3.01%, 26.39% ± 2.22% and 12.20% ± 1.01%, respectively (Figure 3B).

### 3.4 Isorhamnetin regulated apoptotic protein expression in isolated rat hearts subjected to I/R

**FIGURE 4:**
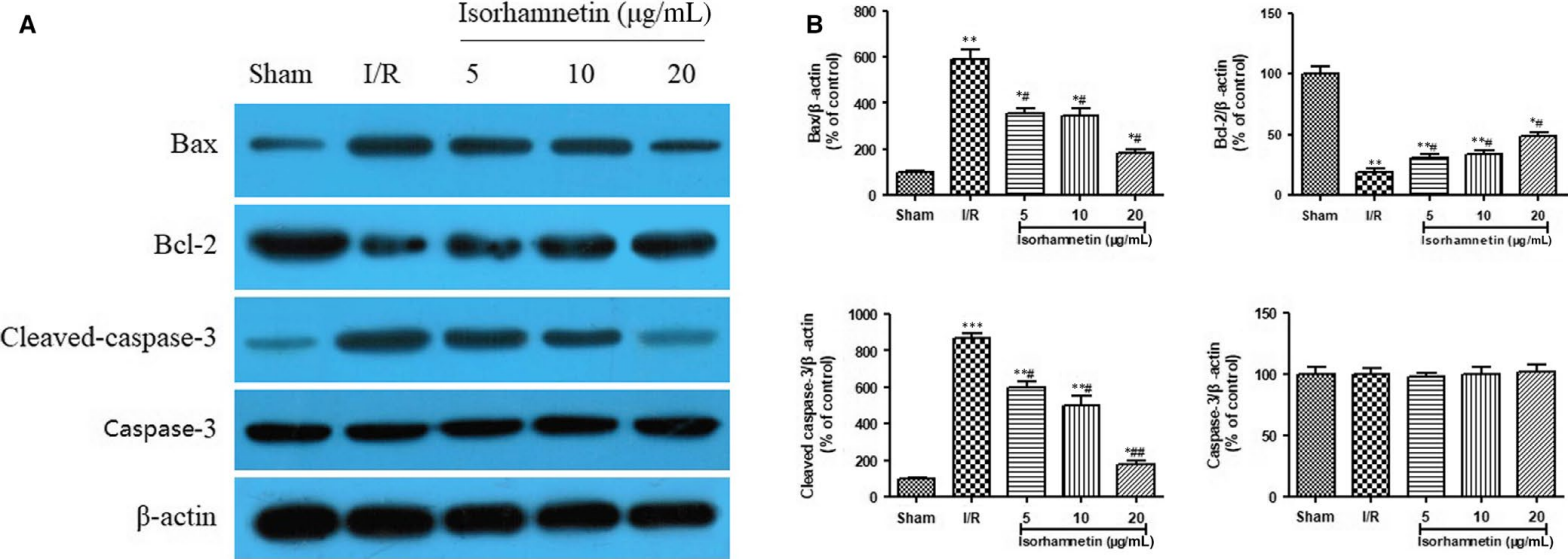
Isorhamnetin regulate the expression of Bax, Bcl‐2 and cleaved‐caspase‐3 protein in isolated rat hearts subjected to I/R. (A) Western blotting analysis of Bax, Bcl‐2 and cleaved‐caspase‐3 protein. (B) Quantitative results of Western blotting analysis. The data were expressed as mean ± S.D (n = 7). ^#^ *P* < .05 and ^###^ *P* < .001 compared with the I/R group; *P < .05, **P < .01 and ***P < .001 compared with the sham group

To confirm the involvement of isorhamnetin on reducing cardiomyocytes apoptosis, the expression of the apoptotic regulatory protein (Bax, Bcl‐2 and cleaved caspaase‐3) in the same part rat of heart tissues was evaluated by Western blot analysis (Figure 4). In agreement with the TUNEL data, the Bax and cleaved‐caspase‐3 protein expression in the I/R‐treated rat hearts were typically enhanced, but the Bcl‐2 protein expression kept at a lower level in comparison with sham group (*P* < .01 or *P* < .001). Interestedly, reperfusion with isorhamnetin (5, 10 and 20 μg/mL) for 45 minutes dramatically increased the level of Bcl‐2 protein and attenuated the protein expression levels of Bax and cleaved‐caspase‐3 compared with the hearts from I/R group (*P* < .05 or *P* < .01). These findings indicated that the apoptosis induced by I/R was remarkably reduced by isorhamnetin.

### 3.5 Isorhamnetin attenuated oxidative stress in isolated rat hearts subjected to I/R

**FIGURE 5:**
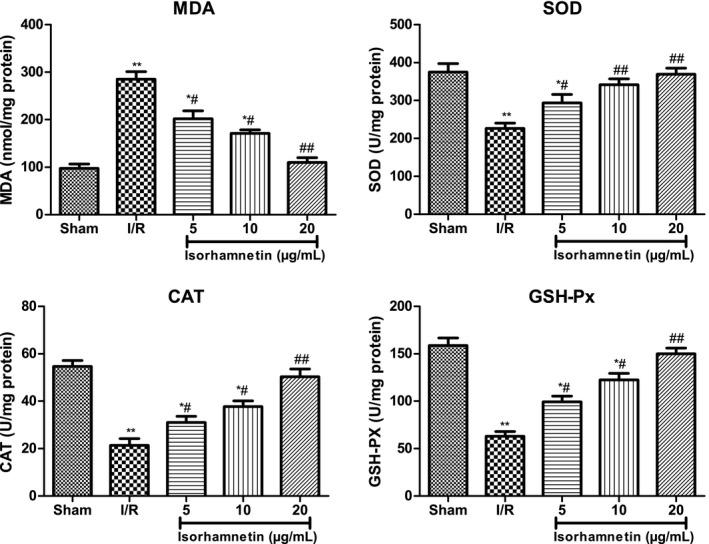
Isorhamnetin ameliorated oxidative stress in isolated rat hearts subjected to I/R. (A) Effects of isorhamnetin on MDA level. (B) Effects of isorhamnetin on SOD activity (C) Effects of isorhamnetin on CAT activity (D) Effects of isorhamnetin on GSH‐Px activity. The data were expressed as mean ± S.D (n = 7). ^#^ *P* < .05 and ^##^ *P* < .01 compared with the I/R group; *P < .05 and **P < .01 compared with the sham group

Oxidative stress is one of the important factors in myocardial I/R injury. We further opted to measure the level of oxidative stress in isolated rat hearts subjected to I/R injury (Figure 5A‐D). MDA level was higher in myocardium of I/R mice (*P* < .01), whereas SOD, CAT and GSH‐Px activities kept in a lower level versus sham hearts (*P* < .01). Interestingly, Isorhamnetin‐treated hearts showed a decreased oxidative level upon I/R injury, as showed by the decreased MDA level and increased antioxidant enzymes activities (SOD, CAT and GSH‐Px) when compared to I/R control hearts (*P* < .05 or *P* < .01).This result suggested that isorhamnetin plays a cardioprotective role by regulating oxidative stress.

## 4 DISCUSSION

The present study is the first, to the best of our knowledge, to examine the cardioprotective effects of isorhamnetin against MI/R myocardial I/R injury in isolated heart infarct model and explore the underlying mechanisms. The doses of isorhamnetin (5, 10 and 20 μg/mL) were chosen based on our preliminary experiment, and the details were not list here. This work demonstrated that isorhamnetin treatment could ameliorate myocardial I/R injury (such as reduction of infarct size and/or cellular apoptosis) in a dose‐dependent manner concomitant with the increased levels of haemodynamic parameters (LVDP, ±dp/dtmax and CF) and anti‐apoptotic protein (Bcl‐2), as well as decreased expression of pro‐apoptotic protein (Bax and cleaved‐caspase‐3).

Cardiac histopathological change was first observed by H&E staining, which was the most widely used to visualize detailed view of the tissue under a microscope.^33^ The H&E staining result demonstrated I/R injury induced disarrangement of muscle fibres, coagulated necrosis, interstitial oedema and large areas of necrosis in isolated rat cardiac tissue subject to I/R as compared with sham control group. These pathological alterations were markedly suppressed by isorhamnetin reperfusion at 5, 10 and 20 μg/mL. Myocardial infarction size was then measured by TTC staining that was normally used as a popular and important parameter to assess the cardioprotective effect of therapeutic drug.^34^ Consistent with the myocardial morphological change, the myocardial infarction area in the I/R group was significantly enlarged when compared to that in the sham group, whereas isorhamnetin treatment (5, 10 and 20 μg/mL) completely shrink the infarction size of cardiac tissues, as determined by TTC staining. The above histopathological improvement and infarction size decrease indicated possible protective effect of isorhamnetin on myocardial I/R injury.

CK and LDH are two classical cytosolic enzymes, serving as biomarkers for the diagnosis of myocardial damage.^35^ In the normal physiological state, they constitutively retain in endochylema of cardiomyocytes. When the cell membrane becomes permeable or destroyed during myocardial IR injury, CK and LDH can easily transit through cytoplasmic membrane and released into the blood. Thus, their activity indirectly represents the extent of myocardial damage.^36^ Next, we measured the release of LDH and CK to evaluate the degree of myocardial injury. Compared with sham‐operated hearts, LDH and CK levels significantly increased in serum of different groups in response to I/R treatment. This increase was effectively suppressed in isolated rat hearts suffering isorhamnetin treatment in a dose‐dependent manner. In line with this result, decreased LDH and CK levels were adopted as the important benchmarks for evaluating the cardioprotective effect of potential drug in many literatures.^30^, ^36^

As we know, for ischaemic heart diseases, reperfusion of the ischaemic myocardium may lead to cardiac dysfunction and eventually cardiomyocyte apoptosis.^37^, ^38^ Hence, improving cardiac function and inhibiting apoptosis is crucial for treating ischaemic heart diseases. We further examined the effect of isorhamnetin on the I/R‐induced cardiac dysfunction in isolated rat heart by monitoring five haemodynamic parameters including LVDP, ±dp/dtmax, HR and CF, which are important indices of cardiac function.^39^ I/R group showed significant decrease of these parameters, except HR, as compared with those in sham group. However, during the course of reperfusion 15, 30 and 45 minutes, isorhamnetin treatment dramatically increased the levels of these haemodynamic parameters as compared to I/R group in a dose‐dependent manner. Intriguingly, the similar results were also identified by Wang et al when they evaluated cardioprotective effect of the xanthones from Gentianella acuta against myocardial I/R injury in isolated rat heart.^39^ Subsequently, the effects of isorhamnetin on cardiomyocyte apoptosis in isolated rats’ hearts subjected to I/R was measured using TUNEL staining. This staining under optical microscopy highlighted the obvious increase of apoptosis in I/R group compared with that in the sham group; the percentage of TUNEL‐positive cells decreased considerably in groups upon treatment with isorhamnetin at doses of 5, 10 and 20 μg/mL. These results suggested that isorhamnetin treatment can alleviate cardiac dysfunction and attenuate cardiomyocyte apoptosis.

Previous evidence indicated that apoptosis was controlled by many factors, such as the Bcl‐2 family, which include pro‐apoptotic Bax protein and anti‐apoptotic Bcl‐2 protein.^40^ They are believed to have the ability to generate mitochondrial membrane permeability, thus allow release of cytochrome c to activate the pro‐apoptotic caspase‐9 and ultimately caspase‐3, resulting in induction of apoptosis.^41^, ^42^ Consistent with this notion, there has been a growing body of evidence to suggest that the up‐regulation the Bcl‐2 and cleaved‐caspase‐3 protein expression level and the down‐regulation of the Bax protein expression level play important roles in protecting heart against I/R injury.^43^, ^44^ In view of this condition, their protein expressions in the rat heart tissues were determined using western blot analysis. The present study demonstrated that I/R resulted in a marked increase of Bax and cleaved‐caspase‐3 protein expression, as well as a marked reduction of Bcl‐2 protein level. Interestedly, this change was eventually reversed in rat hearts tissues following treatment of 5, 10 and 20 μg/mL of isorhamnetin. However, caspase‐3 protein expression remained unchanged in all groups. These results suggested that isorhamnetin could modulate the protein expression of Bax, Bcl‐2 and cleaved‐caspase‐3 to blockade cardiomyocyte apoptosis.

Myocardial I/R injury is a complicated pathophysiological process in which oxidative stress serve crucial roles, thus oxidative stress inhibition is recognized as an important approach to treat I/R‐induced cardiac injury.^45^, ^46^ Wang et al reported that xanthones from *Gentianella acuta* alleviate myocardial I/R injury in isolated rat heart via attenuation of oxidative stress.^39^ It was also reported that attenuation of oxidative stress contributed to the cytoprotective effect of gypenosides against myocardial I/R injury.^47^ Furthermore, we evaluated the effect of Isorhamnetin on oxidative damage in isolated rat hearts subjected to myocardial I/R injury by assessing the SOD, CAT and GSH‐Px activities, as well as MDA level. I/R treatment caused a significant increase in MDA level, but an evident decrease in SOD, CAT and GSH‐Px activities, indicating an increased oxidative level. However, isorhamnetin treatment could efficiently reverse this change in an opposite manner, indicating that anti‐oxidative may play a central role in the cardioprotective effect of isorhamnetin in protecting the heart against I/R‐induced oxidative stress injuries to hearts.

## 5 CONCLUSIONS

In conclusion, our findings provided the first evidence that isorhamnetin could alleviate myocardial I/R injury by suppressing apoptosis via attenuating apoptosis and oxidative stress. Therefore, it is suggested that isorhamnetin can be developed as a promising therapeutic agents for the treatment of myocardial I/R cardiovascular diseases. However, Langendorff isolated heart model, as an ex vivo model, has its limitations. For example, the isolated and perfused heart is free of neurohumoral control, although the preparation is simple and reproducible, and enables the study of the heart without intervention by other organ systems. Next, further studies are still needed to investigate the precise mechanism by which isorhamnetin protects the heart against myocardial I/R injury in vivo and confirm whether isorhamnetin can be used in a clinical setting.

## CONFLICT OF INTERESTS

The authors confirm that there are no conflicts of interest.

## AUTHOR CONTRIBUTION

Yan Xu, Chun Tang, Shengyu Tan, Juan Duan and Yu Yang were involved in conception, design, statistical analysis and drafting of the manuscript. Shengyu Tan and Hongmei Tian contributed in data collection. All authors approved the final version for submission.

## Data Availability

All data generated or analysed during this study are included in this article.
